# Bioactive glasses promote rapid pre-osteoblastic cell migration in contrast to hydroxyapatite, while carbonated apatite shows migration inhibiting properties

**DOI:** 10.1038/s41598-023-47883-2

**Published:** 2023-11-23

**Authors:** Karoliina Kajander, Saara V. Sirkiä, Pekka K. Vallittu, Terhi J. Heino, Jorma A. Määttä

**Affiliations:** 1 Institute of Biomedicine, Faculty of Medicine, University of Turku, Kiinamyllynkatu 10, 20520 Turku, Finland; 2 Department of Biomaterials Science and Turku Clinical Biomaterials Centre – TCBC, Institute of Dentistry, University of Turku, Lemminkäisenkatu 2, 20520 Turku, Finland; 3 Wellbeing Services County of Southwest Finland, Turku, Finland

**Keywords:** Cell migration, Biomaterials

## Abstract

Different biomaterials have been clinically used as bone filling materials, although the mechanisms behind the biological effects are incompletely understood. To address this, we compared the effects of five different biomaterials: two bioactive glasses (45S5 and S53P4), hydroxyapatite (HAP), carbonated apatite (CAP), and alumina on the in vitro migration and viability of pre-osteoblastic cells. In addition, we studied the effects of biomaterials’ calcium release on cell migration, viability and differentiation. We found differences between the materials as the bioactive glasses promoted rapid pre-osteoblastic cell migration. In contrast, CAP decreased cell migration, which was also associated with lower activity of migration related kinases. Bioactive glasses released significant amounts of calcium into the media, while CAP decreased the calcium concentration. The response of cells to calcium was mechanistically studied by blocking calcium sensing receptor (CaSR) and ATP-gated ion channel P2X7, but this had no effect on cell migration. Surprisingly, HAP and CAP initially decreased cell viability. In summary, bioactive glasses 45S5 and S53P4 had significant and long-lasting effects on the pre-osteoblastic cell migration, which could be related to the observed calcium dissolution. Additionally, bioactive glasses had no negative effects on cell viability, which was observed with HAP and CAP.

## Introduction

Biomaterials are classified either according to different biomaterial generations or according to their bioactivity. The hallmark of bioactivity is the capability to form a bond between the material and tissue^1^, which in bone is achieved by mineralization and by inducing osteogenic cells to form bone. Bioactive glasses (BGs, e.g., 45S5 and S53P4) are bioactive, biocompatible, osteoconductive and osteoinductive and can be used to enhance bone formation^2,3^. Their properties are affected by dissolution and interfacial reactions on BG surfaces, where ion exchange reactions alter surface properties of the glass immediately upon contact with tissue fluid^4^.

BGs have been shown to affect bone cells in vitro and in vivo*.* For example, BGs 45S5 and S53P4 can enhance the osteogenic differentiation of immortalized human adipose derived mesenchymal stem cells (MSCs) compared to biologically inert alumina (Al_2_O_3_)^5^. 45S5 and S53P4 are used in several clinical applications and are few of the BGs approved by FDA and EMA for clinical use in humans^3,6^. BG S53P4 is clinically used in implants to repair large calvarial bone defects and it has been radiologically shown to induce remarkable new bone formation^7,8^. BGs are also used due to their antimicrobial properties, which are caused by local increase of pH and osmotic pressure^9^.

Human bone contains carbonated apatite (CAP), while synthetic hydroxyapatite (HAP) is otherwise similar but lacks carbonate^10^. Both HAP and CAP are resorbable materials and possess bioactive and osteoconductive properties, but CAP has been shown to display more osteoconductivity than HAP^10^. On the other hand, bioinert materials, such as alumina, are biostable and biocompatible and possess good mechanical and corrosion resistance properties, while they do not demonstrate bioactivity^11^.

Cell migration is an important process during skeletal development and bone remodeling and healing^12^. During bone turnover, different chemotactic factors are released for example by bone resorbing osteoclasts, which guide osteoblasts and their precursors to the site of function^13^. Upon reconstruction of bone defects by implant, chemotaxis is required to attract cells to the implant site, but to our knowledge, this area is not yet extensively studied.

Calcium has been shown to be one of the major chemoattractants to MSCs, which are capable of differentiating into osteoblasts at the implant site^14^. Osteoblastic cells have different ways of sensing extracellular calcium concentration, for example via the calcium sensing receptor (CaSR), which has been associated with cell migration^15,16^. Calcium can also affect bone cells indirectly, for example via ATP-gated P2X7 receptor (P2X7R)^17^. Osteopontin (OPN) is another factor indicated in the migration of several cell types^18–20^, while its role in osteoblast migration is still incompletely understood.

In this study, we compared the effects of five different clinically used biomaterials: two bioactive glasses (45S5 and S53P4), hydroxyapatite, carbonated apatite and alumina on the migration of pre-osteoblastic cells in vitro and aimed to elucidate the potential migratory mechanisms occurring between biomaterials and cells.

## Results

### Bioactive glasses promote early pre-osteoblastic cell migration while CAP has an inhibitory effect

**Figure 1:**
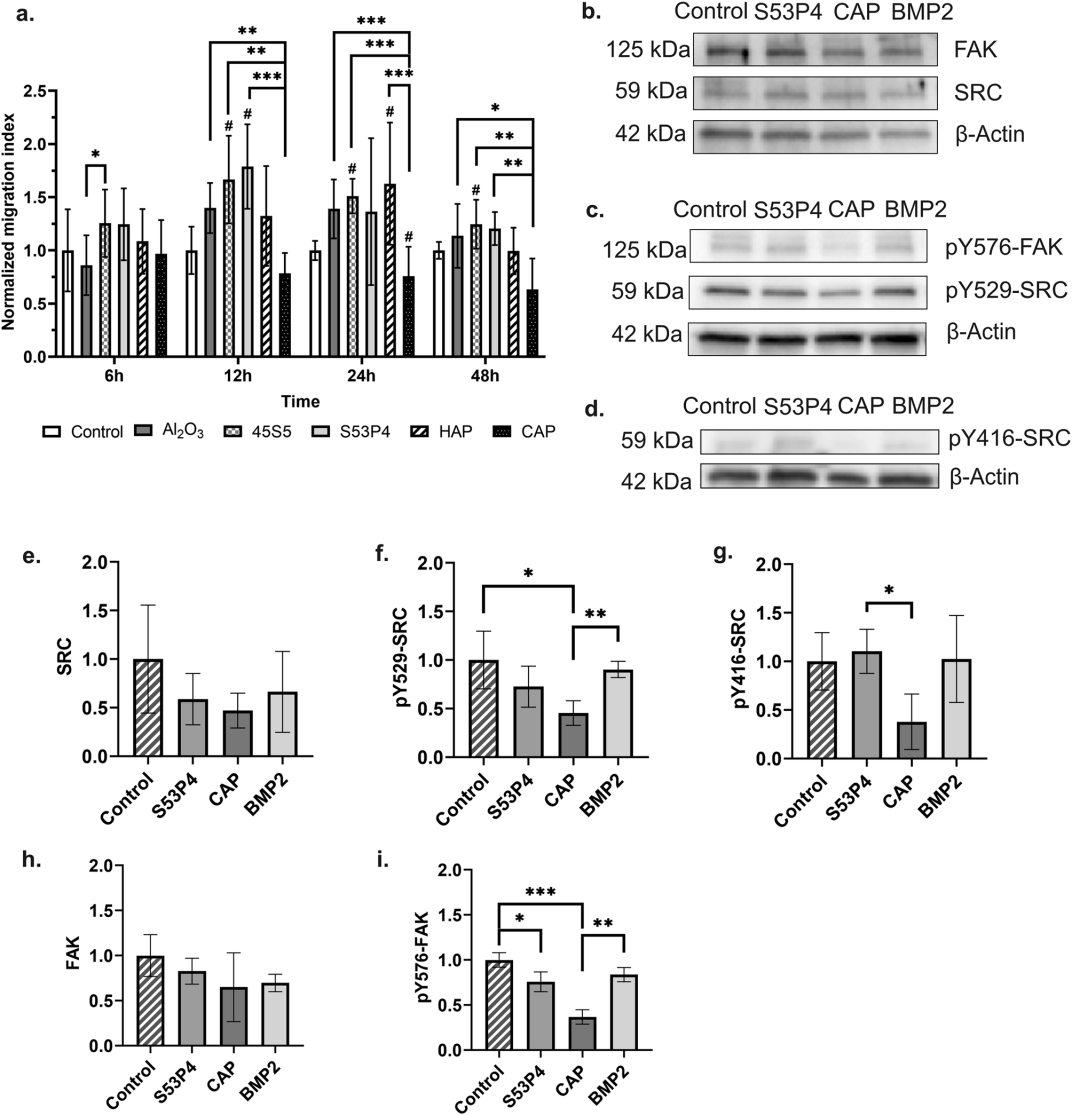
Migration of MC3T3-E1 pre-osteoblastic cells in response to different biomaterials and the expression levels of migration related proteins. (**a**) Number of migrated cells in the Boyden chamber assay at different time points. Results are shown as normalized to control within each time point. (**b**–**d**) Representative cropped images of the protein expression levels of FAK and SRC (**b**), pY576-FAK and pY529-SRC (**c**), and pY416-SRC (**d**) as detected by Western blot. Each figure (**b**–**d**) is from a separate membrane. Full membranes are available in Supplementary figure S4. (**e**–**i**) Quantitative analysis of protein bands is displayed for SRC (**e**), pY529-SRC (**f**), pY416-SRC (**g**), FAK (**h**), and pY576-FAK (**i**), where the control group was assigned as value of 1 within each blot. All data are presented as the mean ± standard deviation of three independent experiments. Parametric data was analyzed using unpaired t-test with Welch’s correction for unequal variances where applicable, and non-parametric data using Mann–Whitney test. Statistical significance is referred to as ^#^p ≤ 0.05 between control and experimental group, and as *p ≤ 0.05, **p ≤ 0.01, and ***p ≤ 0.001 between indicated groups. *HAP* hydroxyapatite, *CAP* carbonated apatite, *FAK* focal adhesion kinase, *BMP2* bone morphogenetic protein 2.

Cell migration was studied in response to different biomaterials using the Boyden chamber assay. At 6 h, there were no major differences in cell migration, except between Al_2_O_3_ and 45S5 (p = 0.011) (Fig. 1a). At 12 h, significantly more cells had migrated towards the wells with BGs 45S5 (p = 0.018) and S53P4 (p = 0.015) when compared to control. At the same time point, significantly more cells had also migrated towards Al_2_O_3_ (p = 0.004) and both BGs (45S5 and S53P4, p = 0.001 for both) when compared to CAP. At 24 h, more cells had migrated towards BG 45S5 (p = 0.047) and HAP (p = 0.024) when compared to control. CAP seemed to inhibit cell migration at 24 h time point, as more migrated cells were seen in Al_2_O_3_ (p < 0.001), BG 45S5 (p = 0.001), HAP (p < 0.001), and control (p = 0.033) groups compared to CAP. However, no statistical significance was reached between BG S53P4 and CAP, probably due to the substantial variation within S53P4 group. At 48 h, only BG 45S5 group differed from the control (p = 0.046). Still, more cells had migrated towards Al_2_O_3_ (p = 0.032), and BGs 45S5 (p = 0.003) and S5P4 (p = 0.006) when compared to CAP. Taken together, both bioactive glasses promote early cell migration, while with HAP the number of migrated cells reached comparable numbers after 24 h, indicating slower migration. CAP was the only material that inhibited cell migration at a consistent trend throughout the whole experiment (Fig. 1a). In all groups, including control, the higher increase in cell numbers was between 24 and 48 h. Absolute cell numbers in migration experiments are available in Supplementary Table S1.

Since BGs promoted cell migration, while CAP showed an overall inhibitory effect, we investigated the phosphorylation state of migration related focal adhesion kinase (FAK) and proto-oncogene tyrosine-protein kinase SRC in response to BG S53P4 and CAP (Fig. 1b–i). BMP2, which is a known chemotactic factor for osteoblasts, was used as a positive control. CAP appeared to decrease the protein levels of studied kinases (Fig. 1e–i), especially pY529-SRC (Fig. 1f) and pY576-FAK (Fig. 1i), in which the difference to control (p = 0.043 and p = 0.0007, respectively) and to BMP2 (p = 0.007) (Fig. 1f) was statistically significant. In addition, BG S53P4 also decreased the level pY576-FAK when compared to control (p = 0.037), but the decrease was much lower than that observed with CAP (Fig. 1i). The levels of pY416-SRC (Fig. 1g) and pY576-FAK (Fig. 1i) were increased in BG S53P4 group compared to CAP (p = 0.026 and p = 0.008, respectively).

**Figure 2:**
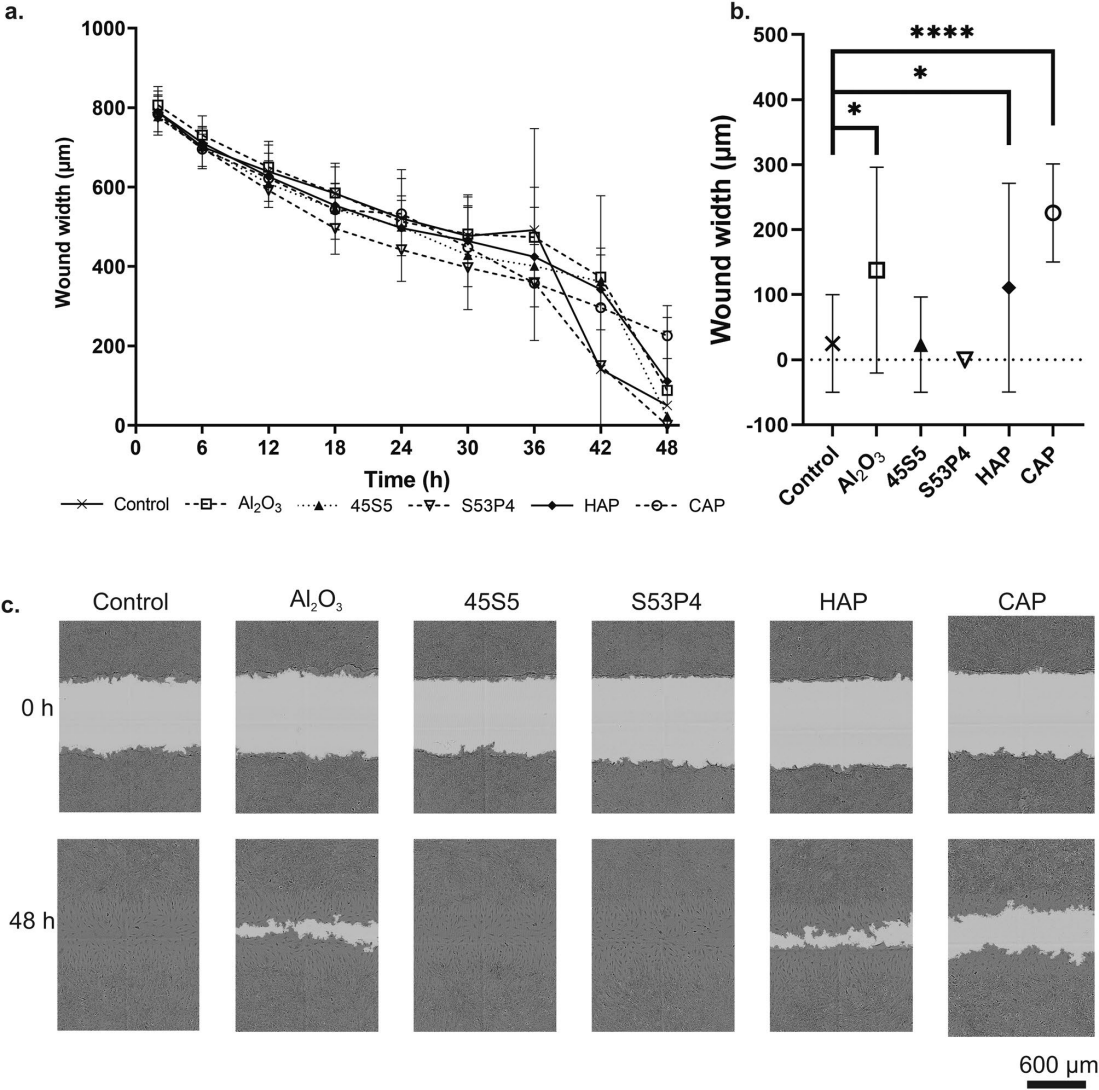
MC3T3-E1 cell migration in the presence of biomaterial-conditioned medium studied with a scratch wound method. (**a**) Wound closure rate by cells in the presence of biomaterial conditioned-media during 48 h. (**b**) Comparisons of wound closure between control and experimental groups at 48 h. (**c**) Representative images of wound areas from each group at the start of the experiment and after 48 h. Data is presented as the mean ± standard deviation of three independent experiments. Non-parametric data was analyzed using Mann–Whitney test. Statistical significance is referred to as *p ≤ 0.05 and ****p ≤ 0.0001. *HAP* hydroxyapatite, *CAP* carbonated apatite.

We also studied cell migration in the presence of biomaterial-conditioned medium with a scratch wound method. In short, a wound was made in a confluent cell layer and then the cells were allowed to migrate into the wound area, the width of which was quantified over time (Fig. 2). After 48 h, the wound was closed both in the control group with normal culture medium and in the groups with BGs S53P4 or 45S5 conditioned medium (Fig. 2a), while the wound closure was incomplete in groups with Al_2_O_3_ (p = 0.012), HAP (p = 0.048), or CAP (p < 0.0001) conditioned medium when compared to control (Fig. 2b). Taken together, our results show that bioactive glasses S53P4 and 45S5 stimulate early pre-osteoblastic cells migration and motility, while CAP has an inhibitory effect.

**Figure 3:**
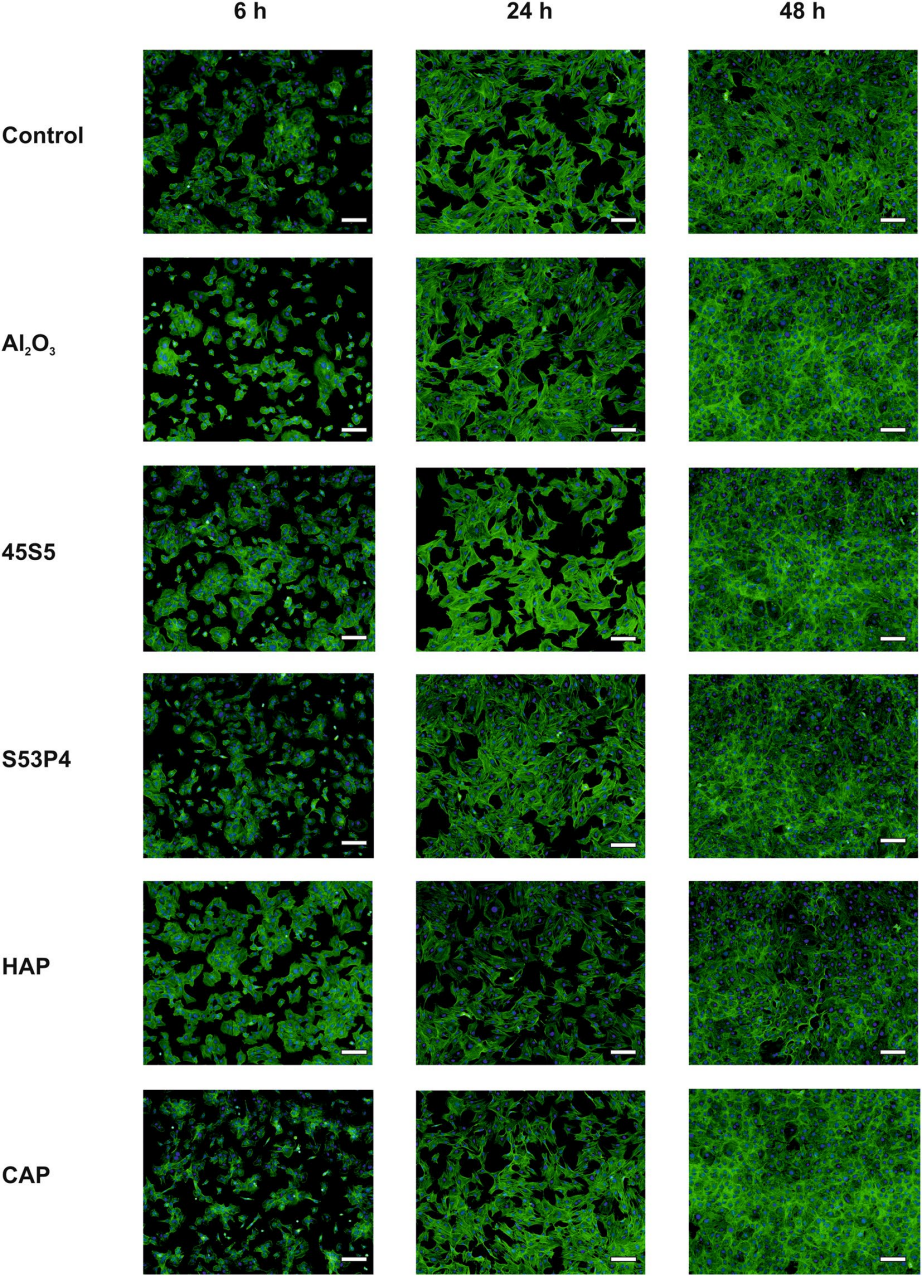
Morphology of MC3T3-E1 cells grown in biomaterial-conditioned medium for 6, 24, or 48 h. The cytoskeleton is visualized by staining the intracellular F-actin with phalloidin (green) and the nucleus with DAPI (blue). Scale bar is 100 µm.

As biomaterial-conditioned media affected cell motility, we also studied potential effects on cell morphology during 48 h by visualization of actin cytoskeleton with fluorescent phalloidin staining (Fig. 3). At 6 h, there seemed to be small differences between groups, since cells grown in media conditioned with S53P4, Al_2_O_3_ or CAP appeared to be smaller and less spread out. However, the differences were attenuated by 24 h, and by 48 h all groups exhibited similar confluent morphology.

### Bioactive glasses and CAP have opposite effects on calcium concentration in cell culture media

**Figure 4:**
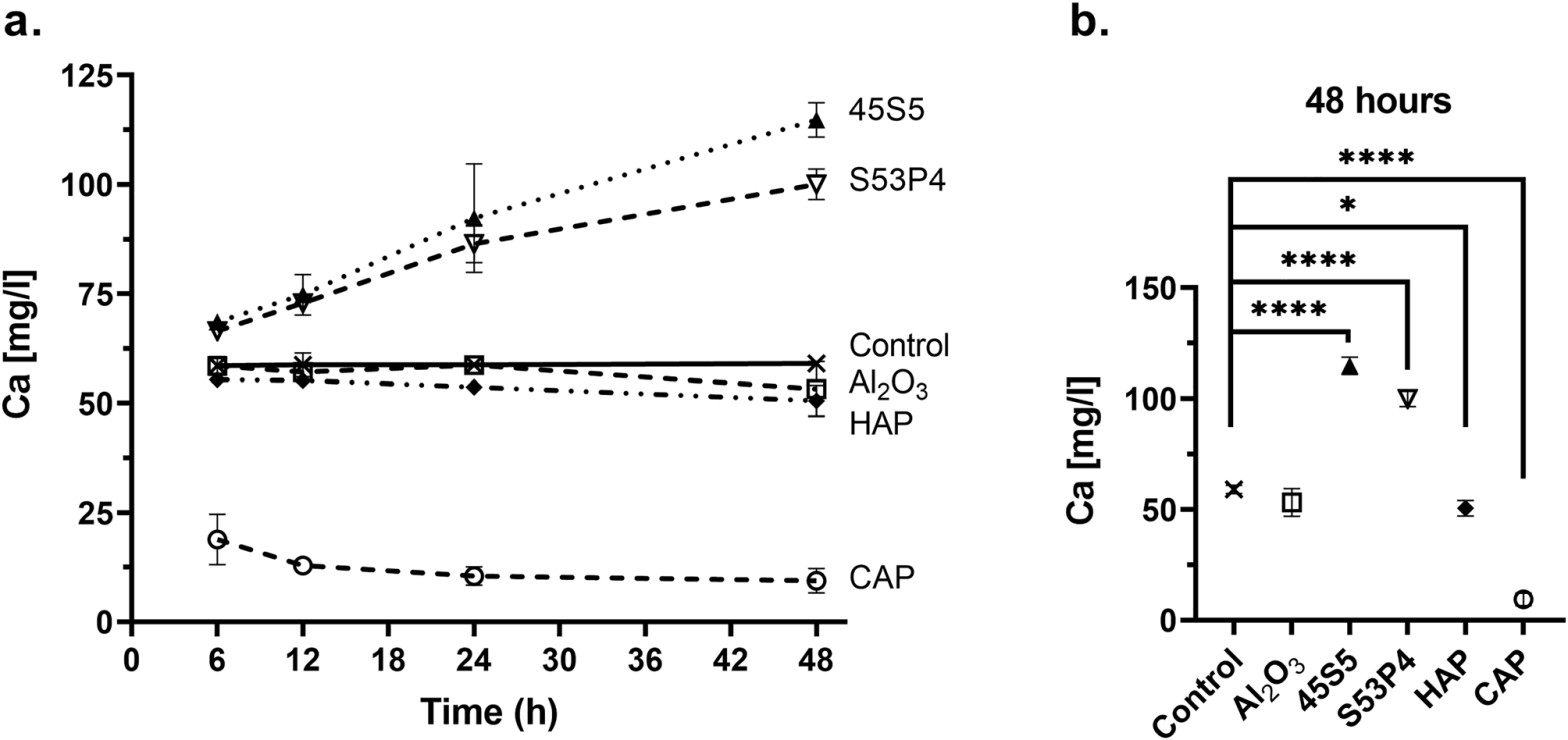
Calcium concentration in the media collected from the migration experiment. (**a**) The change of calcium concentration in the presence of different biomaterials and cells at the culture time endpoints. (**b**) Comparisons of calcium concentrations in different material groups vs control at 48 h. Data is presented as the mean ± standard deviation of three independent experiments. Non-parametric data was analyzed using Mann–Whitney test. Statistical significance is referred to as *p ≤ 0.05and ****p ≤ 0.0001. *HAP* hydroxyapatite, *CAP* carbonated apatite.

Calcium ion concentrations were measured with AAS from media samples collected from migration experiments. The calcium ion concentrations increased steadily in the presence of BGs 45S5 and S53P4 while on the contrary with CAP, calcium concentration remained significantly lower throughout 48 h (Fig. 4a). By 48 h, the calcium ion concentration was significantly increased in the groups with BGs 45S5 (p < 0.0001) and S53P4 (p < 0.0001) compared to control (Fig. 4b). On the contrary, there was a substantial decrease in the CAP group compared to control (p < 0.0001) and a slight, yet significant, decrease in the HAP group compared to control (p = 0.021). Bioinert Al_2_O_3_ had no effects on calcium concentrations in the medium.

We also measured the calcium ion concentration in the biomaterial-conditioned media, which were used in the viability experiments. The presence of CAP significantly reduced calcium concentration in media containing either 0.5% or 10% FBS (p = 0.0003 and p = 0.0002, respectively), when compared to control (Table 1). Conditioning of media with either BG 45S5 or HAP also reduced the calcium ion concentration slightly but the effect was statistically significant only in medium containing 0.5% FBS (p = 0.0011 and p = 0.0385, respectively). To conclude, bioactive glasses increased the calcium concentration of cell culture media, while in the presence of CAP, it was significantly decreased.

**Table 1:** Calcium ion concentrations in biomaterial-conditioned media and in control media (no biomaterials) (mg/mL).

α-MEM with	Biomaterial					
	Control	BG 45S5	BG S53P4	HAP	CAP	Al_2_O_3_
10% FBS	63.1 ± 0.7	55.3 ± 6.4	55.8 ± 6.9	57.5 ± 6.7	34.1 ± 3.7***	54.9 ± 6.4
0.5% FBS	55.6 ± 0.4	47.8 ± 1.6**	48.2 ± 2.9	48.2 ± 3.5*	28.9 ± 4.1***	46.2 ± 5.1

Biological repeats n = 3 and technical repeats n = 3.

*FBS* fetal bovine serum, *BG* bioactive glass, *HAP* hydroxyapatite, *CAP* carbonated apatite.

Statistical significances compared to control are referred to as *p ≤ 0.05, **p ≤ 0.01, ***p ≤ 0.001.

### Blocking of calcium sensing receptor (CaSR) or P2X7 receptor (P2X7R) has no effect on cell migration

Since we observed that BGs and CAP had opposing effects on cell migration and that the calcium concentrations were higher in cell cultures with BGs and lower in cultures containing CAP, we aimed to evaluate more closely the possible mechanisms. Cell migration experiments were repeated while blocking the calcium sensing receptor (CaSR) and the ionotropic ATP-gated receptor P2X7 (P2X7R) but blocking of either receptor did not affect cell migration in any experimental group (Supplementary Fig. S1).

### BG S53P4 decreases osteopontin secretion from pre-osteoblastic cells, but cell migration is not osteopontin-dependent

**Figure 5:**
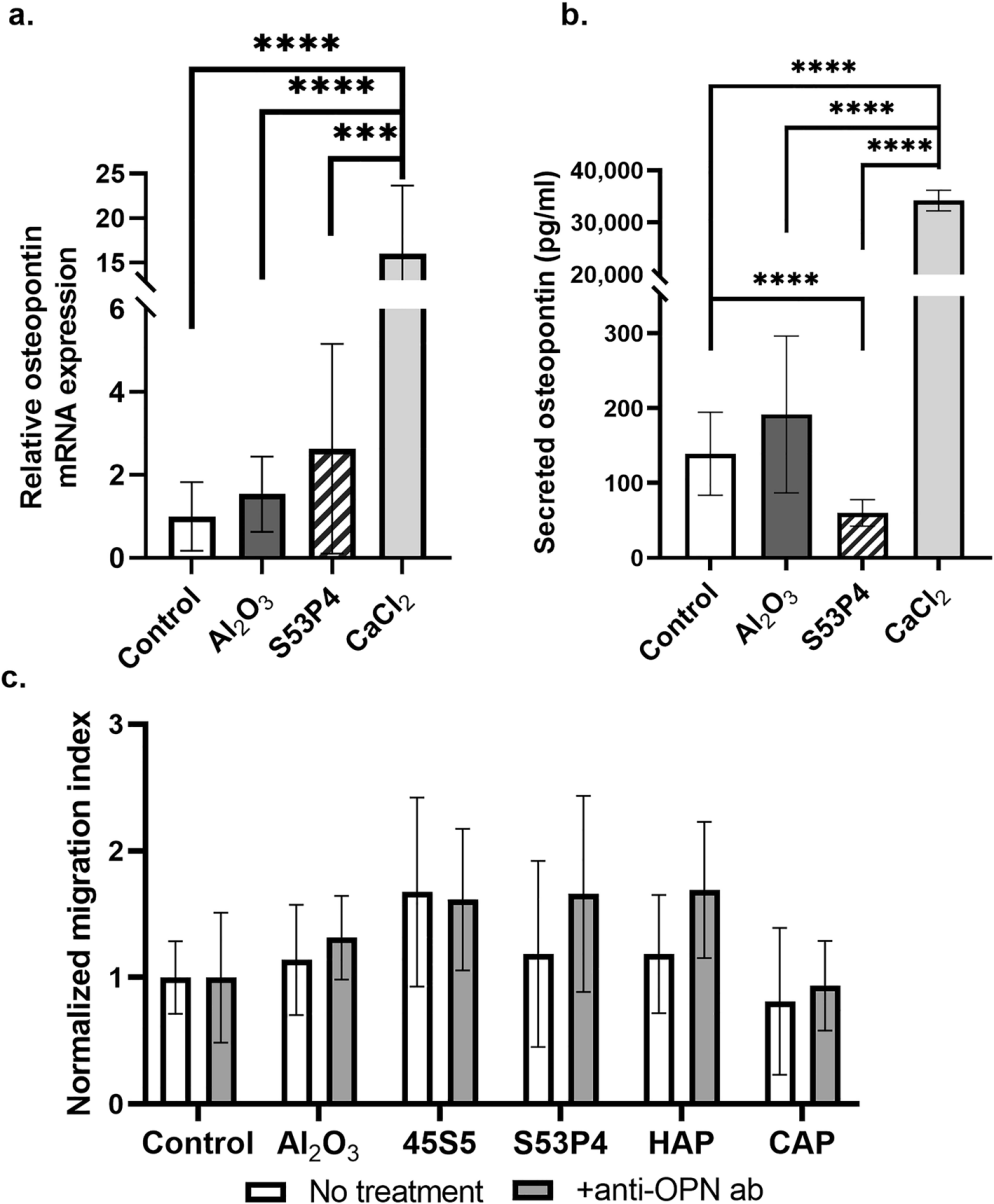
Role of osteopontin in biomaterial-induced cell migration. The expression of osteopontin in MC3T3-E1 cells both at mRNA and protein level in response to S53P4, Al_2_O_3_ or 6 mM CaCl_2_ after 24 h, and the effects of neutralizing anti-osteopontin antibody on cell migration after 12 h. (**a**) The mRNA expression of osteopontin (*Spp1*) and (**b**) the secretion of osteopontin from cells. (**c**) Number of migrated cells in the Boyden chamber assay with neutralizing anti-osteopontin antibody. All data are presented as the mean ± standard deviation of three independent experiments. Statistical significances between control and experimental groups were calculated using Mann–Whitney test for non-parametric data. Statistical significance is referred to as ***p ≤ 0.001 and ****p ≤ 0.0001. *HAP* hydroxyapatite, *CAP* carbonated apatite, *OPN* osteopontin, *ab* antibody.

Since it has been suggested that calcium could affect cell migration via osteopontin^21^, we investigated osteopontin mRNA expression and protein secretion in response to BG S53P4 and Al_2_O_3_. CaCl_2_ at 6 mM is known to stimulate osteopontin expression and was thus used as a positive control. The results demonstrated that after 24 h, 6 mM CaCl_2_ increased the mRNA expression of *Spp1* compared to control, Al_2_O_3_ (p < 0.0001 for both), and BG S53P4 (p = 0.0001) (Fig. 5a). We further observed that cells grown in contact with BG S53P4 secreted less osteopontin compared to control (p < 0.0001) (Fig. 5b), while the osteopontin secretion was increased 245-fold compared to control when cells were exposed to 6 mM CaCl_2_ (p < 0.0001) (Fig. 5b). Similar increase was observed between CaCl_2_ and Al_2_O_3_ (p < 0.0001) and BG S53P4 (p < 0.0001). We also investigated osteopontin secretion from cells in response to calcium sensing receptor (CaSR) and P2X7 receptor blockers after 12 h, but within this time window, no consistent differences were detected in any experimental group (Supplementary Fig. S2).

To further study the role of osteopontin in relation to bioactive materials, we repeated the migration experiment with all the biomaterials of interest in the presence of neutralizing anti-osteopontin antibody. No significant differences were observed between cells treated with osteopontin neutralizing antibody compared to non-treated cells within any group (Fig. 5c). Thus, even though osteopontin levels were increased in response to additional calcium stimulus, no clear effects in relation to cell migration were observed.

### Biomaterials, additional calcium stimulus, or BMP2 do not induce osteoblastic differentiation during the first 48 h

**Figure 6:**
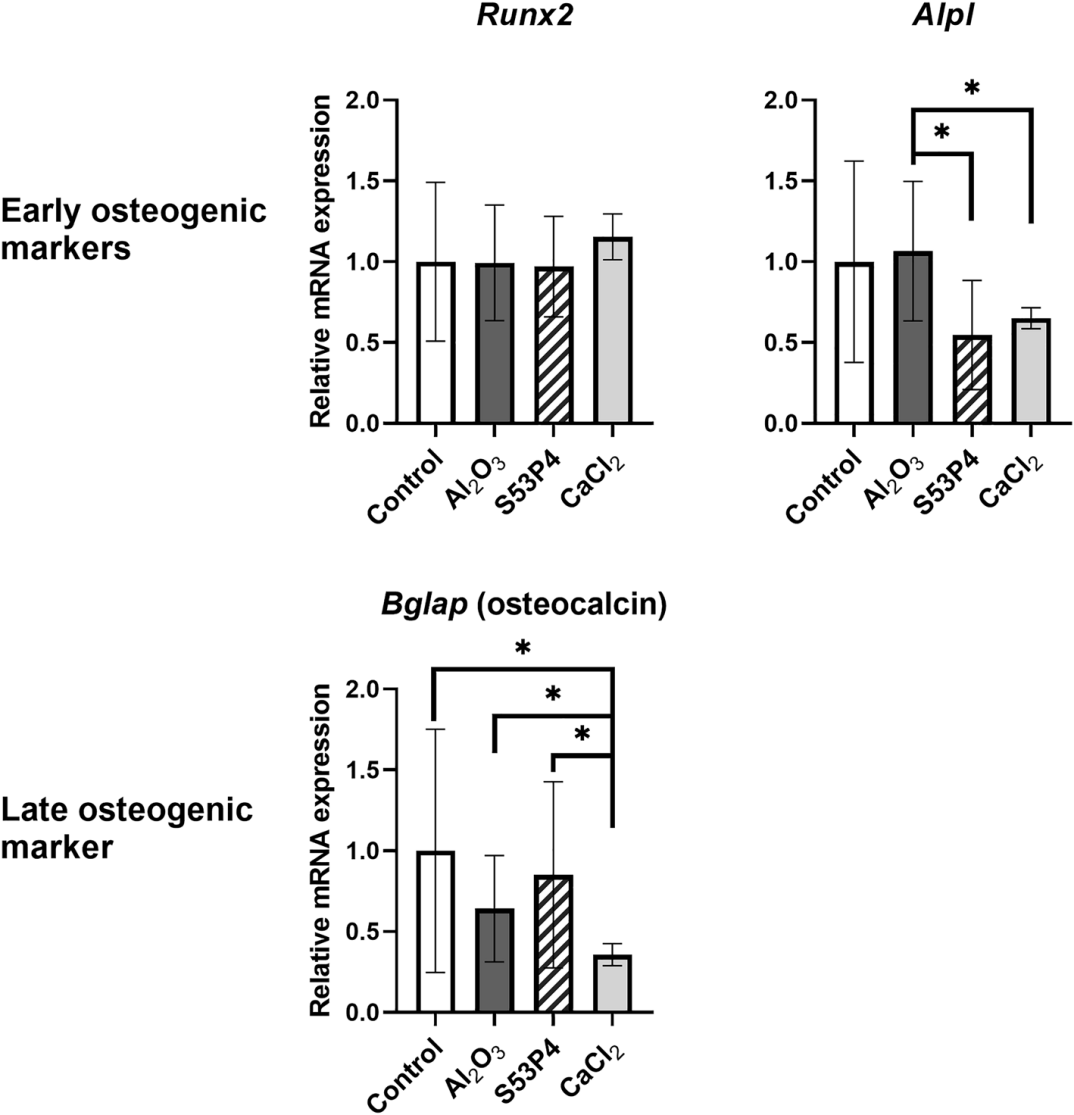
Expression of osteogenic markers. The mRNA expression of early osteogenic marker genes *Runx2* and *Alpl* and late osteogenic marker gene *Bglap* of MC3T3-E1 cells in response to S53P4, Al_2_O_3_and 6 mM CaCl_2_ after 24 h. Results are normalized to control group and all data is presented as the mean ± standard deviation from three independent experiments. Parametric data was analyzed using unpaired t-test with Welch’s correction for unequal variances when applicable, and non-parametric data using Mann–Whitney test. Statistical significance is referred to as *p ≤ 0.05. *Runx2*, runt-related transcription factor 2; *Alpl*, alkaline phosphatase; *Bglap*, bone gamma-carboxyglutamate protein.

Since blocking of calcium-related receptors or the neutralization of osteopontin in the migration experiment did not show uniform effects, we asked whether the observed differences in cell migration could be due to the cells undergoing osteoblastic differentiation. To assess this, we evaluated the mRNA expression of early osteogenic marker genes, i.e., runt-related transcription factor (*Runx2*) and alkaline phosphatase (*Alpl*) as well as a late marker osteocalcin (bone gamma-carboxyglutamate protein, *Bglap*) by qPCR in response to S53P4, Al_2_O_3_, and 6 mM CaCl_2_ after 24 h. The expression of *Runx2* remained unchanged in all groups, but the expression of *Alpl* was lower in S53P4 (p = 0.012) and CaCl_2_ (p = 0.012) groups compared to Al_2_O_3_ (Fig. 6). All treatments showed a trend for decreased expression levels of *Bglap* and the difference was statistically significant between CaCl_2_ group and all other groups (p = 0.011, p = 0.022 and p = 0.032 for control, Al_2_O_3_ and S53P4, respectively) (Fig. 6). Taken together, BG S53P4 and Al_2_O_3_ had no effect on early or late osteogenic gene expression compared to control, suggesting that no notable osteogenic differentiation occurred in MC3T3-E1 cells during the first 24 h of differentiation.

To confirm this result, alkaline phosphatase (ALP) staining was performed on cells grown in the presence of BG S53P4 or CAP. A positive control group was maintained in osteoblastic differentiation medium for 7 days. No ALP-positive cells were observed after 24 or 48 h in any experimental group, indicating that this culture time was not long enough to induce measurable osteogenesis (Supplementary Fig. S3). This is in line with the qPCR data (Fig. 6) showing no changes in osteogenic markers, thus indicating that the observed migratory effects were not differentiation-dependent.

### Cell viability is decreased when cells are cultured in a direct contact with hydroxyapatite or carbonated apatite

We studied the viability of pre-osteoblastic cells both in a direct contact with biomaterials and in the presence of biomaterial-conditioned media with the Alamar Blue method. In addition, both experiments were performed in media with two different FBS concentrations (0.5% or 10%). Cell culture media with 10% FBS was included as a standard culture protocol and cultures with 0.5% FBS were used to evaluate cell viability in the same culture conditions as were used for the migration experiments. In addition, we hypothesized that cell culture media with 0.5% FBS could better reveal any minor effects that biomaterials might have on cell viability compared to cell cultures performed in the presence of 10% FBS, which is rich e.g., in growth factors, vitamins, and proteins and as such could cover biomaterial-induced effects. As expected, when the cells were cultured in media containing 0.5% FBS, lower cell proliferation was observed in all groups, irrespective whether they were in a direct contact with the biomaterials or cultured in biomaterial-conditioned media (Supplementary Fig. S4).

**Figure 7:**
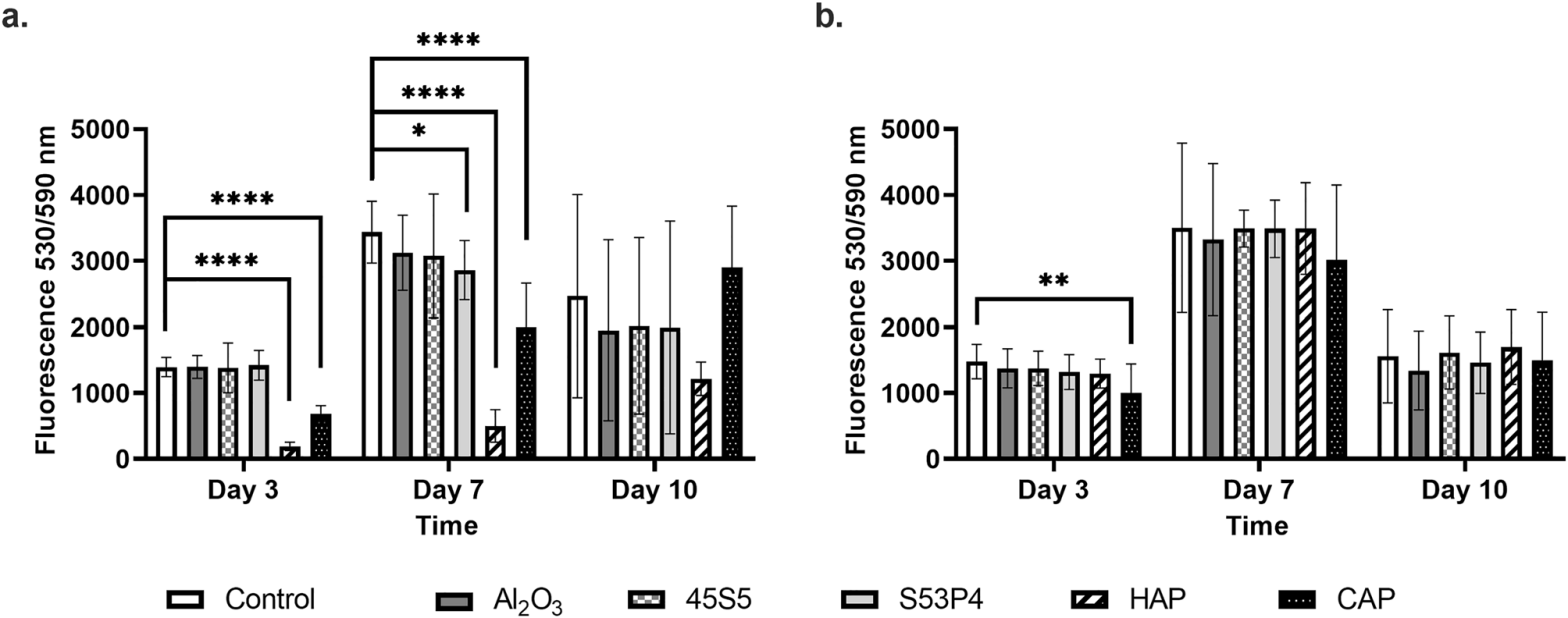
The effects of different biomaterials on the **v**iability of pre-osteoblastic MC3T3-E1 cells in media containing 10% FBS as measured by Alamar Blue. (**a**) Cell viability in the presence of biomaterials. (**b**) Cell viability in the presence of biomaterial-conditioned media. Data is presented as the mean ± standard deviation of three independent experiments. Non-parametric data was analyzed using Mann–Whitney test. Statistical significances are referred to as *p ≤ 0.05, **p ≤ 0.01, ***p ≤ 0.001, and ****p ≤ 0.0001. *HAP* hydroxyapatite, *CAP* carbonated apatite.

In media containing 10% FBS, cell viability was significantly decreased compared to control with both HAP and CAP at days 3 and 7 (p < 0.0001 for all) (Fig. 7a), while slowly recovering towards day 10. In contrast, biomaterial-conditioned media did not significantly affect cell viability, except for the CAP-conditioned group, where cell viability was significantly decreased at day 3 (p = 0.004) when compared to the control group (Fig. 7b). Somewhat surprisingly, cell viability was also significantly decreased in a direct contact with BG S53P4 at day 7 (p = 0.017) (Fig. 7a). In other groups except for HAP and CAP, cell viability was first increased towards day 7, after which it decreased towards day 10. In conclusion, cell viability particularly declined with HAP and CAP when they were in direct contact with the cells.

## Discussion

Different biomaterials have been clinically used as bone filling materials^22^. These materials have different physicochemical properties as they differ in their chemical composition and surface topography and dissolution properties in a liquid environment^22,23^. However, the mechanisms behind the biological effects of bioactive materials are incompletely understood. The current study was therefore conducted to study the effects of two bioactive glasses (45S5 and S53P4), hydroxyapatite, carbonated apatite, and alumina on migration and viability of pre-osteoblastic cells and activation of migration-related signaling proteins.

Boyden chamber migration experiment indicated that both bioactive glasses (BGs) 45S5 and S53P4 as well as hydroxyapatite (HAP) induced a small but significant increase in pre-osteoblastic cell migration towards the materials after 12 and 24 h. Similar phenomenon was not detected with carbonated apatite (CAP), which in contrast showed negative effects on cell migration. Especially in the presence of BGs, the positive effects on cell migration happened earlier when compared to HAP, where the migration appeared to occur slower. The positive effects of bioactive glasses on cell migration were demonstrated also with scratch-wound assay. With this method, the slower migratory effects in the presence of HAP and alumina were also evident, although it is possible that wound closure would occur also in these groups over a longer follow-up time. The reduced cell migration observed with CAP was associated with decreased levels of migration-promoting SRC and FAK kinases. These results indicate that soluble factors released from the bioactive glasses stimulate osteoblastic cell migration and could thus contribute to their osteoinductive properties.

One of the main components and dissolution products of bioactive materials is calcium^10,23–25^, which is essential for normal cell adhesion and migration^26^. We observed an increase in the calcium ion concentration in medium incubated with BGs, while the presence of CAP substantially reduced calcium ion concentration in the medium. The measured static release of calcium from bioactive glasses is in line with previous studies with non-static, fluid-flow experimental setups^23,25^, but the observed calcium lowering effect by CAP was somewhat unexpected. The calcium depleting effect might be explained by the precipitation of apatite, which consumes the calcium, and probably phosphorus from the media^27^. As calcium is readily dissolved from BGs, the medium becomes saturated with it before the formation of apatite-layer onto BGs, which could explain why calcium is not depleted from the medium with BGs.

As BGs increased cell migration in vitro, and as calcium is known to be essential for normal cell adhesion and migration^26^, it is tempting to propose that the observed positive effect could be due to BGs’ calcium-releasing properties. Earlier studies have indeed demonstrated that increased extracellular calcium improves the migration of MC3T3-E1 pre-osteoblasts towards higher calcium concentration in vitro^15^. In order to study if the cells are responding to calcium in our migration experiments, we blocked the calcium sensing receptor (CaSR) or ATP-gated ion channel P2X7, both of which are known to be expressed on MC3T3-E1 pre-osteoblasts^15,28^. However, neither blocking of CaSR nor P2X7 in MC3T3-E1 cells decreased cell migration. Thus, it can be speculated that the migratory effects of calcium may be transmitted via other mechanisms than receptor-mediated calcium signaling. In addition, CaSR is known to have a high turnover, as it is consecutively endocytosed and new CaSR is inserted to the cell membrane^29^. Thus, it is possible that we were unable to completely block the receptor within the experimental time frame. Consequently, we cannot completely exclude its role in the observed migration phenomenon. Furthermore, other physicochemical properties of the materials, such as surface charge and topography may explain the differences in cell migration, since they are known to influence the adsorption of proteins and ions onto the material surface^30^. In support of this, it has been previously shown that proteins and ions adsorbed onto biodegradable materials can enhance the adhesion of osteoblasts onto biomaterials in vitro^22,30,31^. Interestingly, alumina also seemed to increase cell migration, even though it is chemically inert and does not release ions into the surrounding fluid. However, we have previously reported that the alumina applied also in the current study has trace quantities of sodium, calcium and phosphorus, which could potentially dissolve into the surrounding fluid^23^ and thus explain the small increase in cell migration. It is also possible that small amounts of serum protein could precipitate onto alumina’s surface^32^, which might create a migratory gradient for cells^22^.

The migratory indexes between the two studied BGs, S53P4 and 45S5, differed slightly, which might be explained by the fact that these glasses differ from each other in terms of ion release^3,23,25^. BG 45S5 has stronger alkalinity and a quicker ion release than BG S53P4, which in turn induces more long-lasting effects^3,25^ and such differences might affect cell migration. It is also possible, that sterilization by autoclaving might affect the material surface chemistry and contribute to the slight differences between materials. However, the differences between BGs became evident only when they were compared to control, and the measured values between the BGs were rather similar at all time points. Based on this, there actually might not be a considerable biological difference between these two bioactive glasses concerning cell migration.

Our results show that a high calcium concentration (6 mM CaCl_2_) increased osteopontin (OPN) mRNA expression and the amount of secreted protein in MC3T3-E1 pre-osteoblasts. OPN is known to enhance the migration of MSCs from which osteoblasts are derived^19,33^. Furthermore, elevated extracellular calcium has been shown to increase the expression of OPN in MSCs both at mRNA and protein level after 24 h, and it has been suggested that the calcium-induced migration is actually occurring via increased OPN expression^21^. However, even though the cells showed a higher migratory behavior, we did not observe increased OPN expression in cells cultured in the presence of BG S53P4. This suggests that there most likely are other mechanisms and signaling molecules affecting BG induced cell migration, and thus more research in this topic is needed.

Bioactive glasses showed no effects on the expression levels of osteoblastic differentiation markers. However, cell viability was normal in the presence of both BGs until day 7, after which it decreased closer to the values obtained on day 3. The growing number of cells could explain the initial increase but when the dividing cells eventually fill the growth area, it could lead to a subsequent decrease in viability. On the contrary, cell viability was reduced from the very beginning when cells were grown in the presence of HAP or CAP, which is surprising, since both are similar components of bone mineral. When the cells were grown in the presence of HAP, the decrease in cell viability was substantial, which is in line with earlier in vitro observations^34,35^. Similar, yet a weaker effect was caused by CAP, which could be explained by our observation of CAP-induced calcium depletion from the medium. Even though cell viability was initially low with both HAP and CAP, it steadily increased when the cells seemed to recover over time.

In line with these observations, we have recently reported a significant inhibitory effect on cell viability and proliferation with functionalized calcium carbonate, which contains 51% of HAP^36^. HAP particle surface has been demonstrated to be very porous^23^ and according to our observations, including the current study, it can degrade into smaller particles in cell culture conditions. Based on earlier data showing that osteoblastic cells can endocytose HAP particles^34,35^ and functionalized calcium carbonate particles^36^, it is possible that such internalization impairs cell’s metabolic processes and thereby viability. Because of this, it is important to consider the consequences of biomaterials fragility, when these types of materials and their potential effects on cells are studied in vitro.

Even though it is well established, that the ideal bone grafting material should provide osteoinductive factors to recruit bone-forming cells to target location^37^, there is an unmet need to better understand the mechanisms by which biomaterials stimulate cells^38^. In general, our current data shows that there are differences between the biomaterials currently in use concerning their effects on cell migration and viability. Although we were not able to describe the exact mechanisms by which the bioactive materials and calcium are affecting cells, our results suggest that receptor-mediated calcium signaling is not the only way by which cells migrate in response to different calcium concentrations. Furthermore, osteopontin, which has previously been linked to calcium-mediated cell migration, might be a less important signaling molecule regarding bioactive glasses.

In conclusion, our current study shows that bioactive glasses 45S5 and S53P4 have a more positive effect on the migration of pre-osteoblastic MC3T3-E1 cells compared to CAP, which inhibited cell migration. HAP also showed a positive trend for cell migration, but the effect happened slower compared to BGs. In addition, both CAP and HAP might, at least in vitro, have adverse effects on cell viability. Even though biomaterials have been successfully used for decades, this is the first study to our knowledge to address the pre-osteoblastic migration and the underlying mechanisms in this setting, which in turn emphasizes the need for further studies within this field.

## Materials and methods

### Biomaterials and preparation of biomaterial-conditioned media

Five different biomaterials with approximately the same particle size were studied: bioactive glass 45S5 (500–800 µm, 24.5 wt% Na_2_O, 45 wt% SiO_2_, 6 wt% P_2_O_5_, 24.5 wt% CaO; manufacturer: MO-SCI Health Care, L.L.C. 4040 HyPoint North, Rolla MO 65401 USA), bioactive glass S53P4 (500–800 µm, 23 wt% Na_2_O, 53 wt% SiO_2_, 4 wt% P_2_O_5_, 20 wt% CaO; manufacturer: Mo-SCI Health Care L.L.C. 4040 HyPoint North, Rolla MO 65401 USA), alumina (500 µm, 99.75 wt% Al_2_O_3,_ 0.25 wt% Na_2_O, 0.02 wt% SiO_2,_ 0.02 wt% Fe_2_O_3;_ manufacturer: Duralum White (Washington mills USA)), hydroxyapatite (100–300 µm, Ca_10_(PO_4_)_6_(OH)_2_; manufacturer: Berkeley advanced biomaterials, INC. 901 Grayson Street, Suite 101, Berkeley, CA 94710), and carbonated apatite (300–600 µm, Ca_10_(PO_4_)_6_(OH)_2_ with carbonate substituting group; manufacturer: Cytrans Granules GC Corporation, Tokyo, Japan). The chemical structure of CAP depends on carbonate substituting group; in type A, carbonate is substituting for OH^-^ and in type B, for PO_4_^3−^. The CAP used for this study contained both type A and type B. For detailed data on characterization of the materials, see Sirkiä et al.^23^.

For all cell culture studies, the biomaterials were first weighed on aluminum foil and then sterilized in the autoclave (+ 121 °C, 20 min). For experiments with biomaterial-conditioned media, the media was prepared by dissolving each biomaterial in cell culture media at 37 °C, 5% CO_2_ for 24 h. After dissolving, conditioned media was collected, centrifuged, and decanted in a new tube and used for cell culture.

### MC3T3-E1 cell cultures

MC3T3-E1 (subclone 4) pre-osteoblastic cells (ATCC, CRL-2593™) were cultured in phenol-red free αMEM (Gibco, 41061029), supplemented with fetal bovine serum (FBS, 10%, Gibco, 10270106) and Penicillin–Streptomycin (1%, Gibco, 15140148). For osteoblastic differentiation, medium was further supplemented with dexamethasone (10^–8^ M, Sigma, D4902), sodium beta-glycerophosphate (10 mM, Fluka, 50020), and ascorbic acid-2-phosphate (70 µg/mL, Sigma, A8960). The cells were maintained at + 37 °C, 5% CO_2_, and the growth medium was changed every 2 to 3 days. Cells between passages 4–11 were used for experiments.

### Cell migration assay

The effect of different biomaterials on cell migration was examined using a Boyden chamber assay, where the chemotactic attraction of cells can be tested by placing them in the perforated upper chamber and to evaluate their migration towards the materials in the lower chamber. First, 15 mg of each biomaterial was placed at the bottom of a 24‑well plate well and 700 μL of medium with 0.5% FBS was added. The lower FBS concentration was used to minimize the possible effects from the serum. Cell culture inserts with 8.0 µm pores (Falcon, 353097) were placed in the wells and 10,000 cells in 200 μL of medium was added per insert (30,000 cells/cm^2^). After 6, 12, 24, and 48 h the inserts were removed from the wells and fixed with paraformaldehyde (PFA, 4%) for 15 min. At these time points, the medium was also collected for further analysis. After fixation, the un-migrated cells were removed from the top part of the insert using a cotton swab. The nuclei of migrated cells localized underneath the insert were stained with Hoechst 33258 (5 μg/mL) solution for 15 min. The inserts were imaged using EVOS 5000 microscope with 4X magnification capturing approximately 80% of the insert surface. The number of migrated cells was counted using ImageJ software (v1.52p). The number of migrated cells in empty wells represents random movement of the cells in the system and these wells were considered as controls for each time point. The experiment included three technical replicates for each condition and each experiment was repeated three times.

### Scratch wound assay

Cells were plated into 96-well ImageLock plates (30,000 cells/cm^2^) in 100 µL growth medium. After 24 h, scratch wounds were made in the cell monolayer using IncuCyte Woundmaker tool. After this, the wells were washed with phosphate buffered saline (PBS) and the growth medium was changed into medium with 0.5% FBS conditioned either with BG S53P4, BG 45S5, Al_2_O_3_, HAP or CAP. The conditioned medium was prepared by incubating 115 mg of each biomaterial with 5 mL medium, as described above. Medium incubated without any biomaterial served as a control. The wells were automatically imaged by the IncuCyte software every two hours for 48 h with a 10 × objective. Wound width was calculated based on the processed images by IncuCyte Scratch Wound Analysis Software Module. The automatically processed images were manually inspected and incorrectly identified wounds were excluded. The wound closure was further assessed by statistically comparing the end point values of each biomaterial group to control group. The experiment included eight technical replicates for each condition and the experiment was repeated three times.

### Fluorescence staining

The cytoskeleton of MC3T3-E1 pre-osteoblastic cells was visualized by staining the intracellular F-actin with phalloidin. The cells were plated into 8-well glass coverslips (Millicell, PEZGS0816, 30,000 cells/cm^2^) in 200 µL of control medium or biomaterial-conditioned medium (10% FBS). The conditioned medium was prepared by incubating 115 mg of each biomaterial with 5 mL medium as described above. After 6, 24, and 48 h, the cells were fixed in PFA (4%). The cytoskeleton was visualized by staining with Phalloidin-Fluor 488 (Abcam, ab176753) according to the manufacturer’s instructions. Mounting medium with DAPI (Abcam, ab104139) was used to mount the samples and stain the nucleus. The cells were imaged using a fluorescence microscope (Zeiss Axioimager).

### Protein isolation and Western blotting

For protein isolation, MC3T3-E1 cells were plated into 6-well culture plates. First, 76 mg of BG S53P4 or CAP was added to the wells. Then cells in 3 mL medium with 0.5% FBS were seeded into each well (30,000 cells/cm^2^). As a positive control, bone morphogenetic protein 2 (BMP2, 1 ng/mL, R&D Systems™, 355-BEC-010) was added to the cells without any biomaterials. After 24 h, cells were lysed in RIPA lysis buffer supplemented with protease (Pierce™, 88666) and phosphatase (Pierce™, A32957) inhibitors. Protein lysates for each condition were pooled from two replicate plates. Protein concentrations were quantified using BCA Protein Assay kit (Pierce™, 23225). Total protein (15 µg) was run on pre-cast gels (Bio-Rad, 4569033) and electro-transferred to polyvinylidene difluoride (PVDF) membranes, which were blocked with BSA in TBST (5% w/v), followed by incubation with primary antibodies for SRC (Invitrogen, PA5-17717, 1:1000), pY416-SRC (Invitrogen, PA5-97366, 1:1000, pY529-SRC (Invitrogen, 44-662G, 1:1000), FAK (Invitrogen, AHO0502, 1:100) and pY576-FAK (Invitrogen, PA5-104964, 1:750) diluted in TBST. After overnight incubation, the membranes were incubated with goat anti-rabbit HRP-conjugated secondary antibody (Abcam, ab205718, 1:20,000) and HRP-conjugated anti-β-actin (ACTB, Sigma, A3854, 1:50,000) for 1 h at room temperature. The chemiluminescent reaction was induced with SuperSignal™ West Pico PLUS Chemiluminescent Substrate (Thermo Scientific, 34580) and the protein bands were detected using Sapphire™ Biomolecular Imager. Full membranes with all proteins were detected simultaneously using automatic exposure time. Quantitative analysis of the protein bands as gray values was done using ImageJ software (v1.52p). All processing was applied equally across the entire image including controls. Normalization factor was calculated by dividing the housekeeping protein ACTB from reference sample with the ACTB from treated sample. Normalized value for each sample was then calculated by multiplying the band quantity of the protein of interest with the ACTB normalization factor. The experiment was repeated three times. Images of full-length gels chosen for representative images can be found in in Supplementary Fig. S5.

### Calcium release

In vitro concentration of calcium released from the biomaterials was measured with Atomic Absorption Spectroscopy (AAS, PerkinElmer AAnalyst 400 with a Ca-lamp) both from the biomaterial-conditioned mediums and from the medium collected from the migration experiment at 6, 12, 24, 48 h. Media were first diluted with ultrapure water for CAP samples in 1:10 and for others 1:20. After water dilution the samples were mixed with La-solution (5% wt/vol), which was prepared by dissolving La_2_O_3_ with HCl (37%) and then diluted 1:4 with water. The diluted samples were then centrifugated at 2800 rpm for 10 min and Ca concentrations were measured with AAS afterwards. Measurements were repeated four times from biomaterial-conditioned mediums and from all three technical repeats of each migration experiment.

### Calcium sensing receptor (CaSR) and P2X7 receptor blocking

In order to study the role of calcium in cell migration, the migration experiment including all biomaterials of interest was repeated in the presence of calcium sensing receptor (CaSR) blocker and P2X7 receptor (P2X7R) blocker. NPS2143 hydrochloride (1 µM, Sigma, SML0362) was used to block the CaSR and A839977 (500 nM, Tocris, 4232) to block P2X7R. Both reagents were diluted in DMSO, and vehicle controls with the same concentration of pure DMSO were included in the experiments. After 12 h, the inserts were fixed, stained, imaged, and analyzed as described above and the medium was collected for ELISA analysis. The number of migrated cells cultured in the presence of inhibitors was compared to control cells cultured without inhibitors. The experiment included three technical replicates for each condition and the experiment was repeated twice.

### RNA isolation and quantitative PCR

MC3T3-E1 cells were plated into 24-well culture plates in 900 µL medium with 0.5% FBS (30,000 cells/cm^2^) together with 15 mg of BG S53P4, Al_2_O_3_, or 6 mM CaCl_2_ per well. After 24 h, the cells were lysed, and total RNA was extracted using NucleoSpin Mini Kit for RNA Purification (Macherey–Nagel, 740955) following the manufacturer’s instructions. At the same time point, the medium was collected for ELISA analysis. The experiment included three technical replicates for each condition and the experiment was repeated three times.

RNA concentration was determined with NanoDrop One spectrophotometer (ThermoScientific) and RNA was reverse-transcribed into cDNA using High-Capacity cDNA Reverse Transcription Kit (Applied Biosystems, 4368814). 10 ng of cDNA was used for amplification of target genes with qPCR utilizing the Taqman® gene expression assay (ThermoFisher). Peptidylpropyl isomerase B (*Ppib*) was used as a housekeeping gene. Gene expression was quantified using the ΔΔCt method and fold change was calculated with the 2^-ΔΔCt^ formula.

### Neutralization of osteopontin

To study the role of osteopontin, the migration experiment was performed in the presence of osteopontin neutralizing antibody (20 ng/mL, R&D Systems, AF808-SP) diluted in sterile PBS. After 12 h, the inserts were fixed, stained, imaged, and analyzed, as described above. The number of migrated cells cultured in the presence of neutralizing antibody was compared to control cells without antibody treatment. The experiment included three technical replicates for each condition and the experiment was repeated three times.

### Osteopontin (SPP1) ELISA assay

The secretion of osteopontin was quantified using the mouse OPN (SPP1) ELISA kit (Invitrogen, EMSPP1) according to the manufacturer’s instructions. The absorbance was measured at 450 nm using EnSight™ Multimode plate reader (PerkinElmer). The samples for ELISA included media from six replicate experiments that were collected prior to cell lysis for RNA extraction or media from one cell migration experiment with CaSR and P2X7 receptor blocking.

### Alkaline phosphatase staining

To assess osteoblastic differentiation, the cells were stained for alkaline phosphatase (ALP). Briefly, MC3T3-E1 cells (30,000/cm^2^) were plated into 24-well plates in 900 µL medium with 10% FBS together with 15 mg of S53P4 or CAP. A control without materials and a positive control with osteoblastic (OB) differentiation promoting medium were also included. After 24 and 48 h, cells were fixed with PFA (4%) and stained for ALP according to the manufacturer’s instructions (Sigma Diagnostics, 86-R). Another positive control without materials and with OB differentiation promoting medium was cultured for 7 days during which osteogenic differentiation is known to occur, and then fixed and stained as described above. The experiment included two technical replicates for each condition and was performed once.

### Cell viability assay

Viability of MC3T3-E1 pre-osteoblastic cells was assessed in the presence of biomaterials and in the presence of biomaterial-conditioned media with two different FBS percentages (0.5% or 10%). Control cultures were performed in normal cell culture medium with 0.5% or 10% FBS without any biomaterials or biomaterial-conditioned media. In biomaterial experiments, each well contained 15 mg of biomaterial and 900 µL of cell culture media and the biomaterial-conditioned media was prepared with the same percentage of biomaterial (15 mg/900 µL) and as escribed above.

MC3T3-E1 cells were seeded into 24-well plates (5000 cells/cm^2^) in 900 µL culture medium, which was changed every 3–4 days. Cell viability at time points of 3, 7, 10, days was determined by Alamar Blue method by measuring fluorescence at 530/590 Ex/Em (Synergy HT with software Gen5 version 2.09). Background fluorescence in culture medium was measured to represent assay blank and was subtracted from sample values, when viability results were analyzed. Each experimental group contained three technical repeats (wells) and each experiment was repeated three times.

### Statistical analysis

Statistical testing was done using GraphPad Prism version 8.0 (GraphPad Software, San Diego, CA, USA). Comparisons for normally distributed data were performed using unpaired t-test. If the variances between groups were unequal, Welch’s correction was used. Unpaired nonparametric data was analyzed using the Mann–Whitney test. P < 0.05 was chosen as the threshold of statistical significance. Data is shown as the mean ± standard deviation.

### Supplementary Information

Supplementary Information.

## Data Availability

The raw data supporting the conclusions of this article will be made available by the corresponding author Jorma Määttä (jmaatta@utu.fi), at reasonable request.
