# Supplementary material for: Bioactive glasses promote rapid pre-osteoblastic cell migration in contrast to hydroxyapatite, while carbonated apatite shows migration inhibiting properties
Source: Sci Rep. 2023 Nov 23;13:20587. doi: 10.1038/s41598-023-47883-2 (PMC10667509; doi:10.1038/s41598-023-47883-2)
Supplement: Supplementary file 1 — Supplementary Information. [file 41598_2023_47883_MOESM1_ESM.pdf]

Bioactive glasses promote rapid pre-osteoblastic cell migration in contrast to hydroxyapatite, while carbonated apatite shows migration inhibiting properties

Karoliina Kajander[1*], Saara V. Sirkiä[2], Pekka K. Vallittu[2,3], Terhi J. Heino[1], and Jorma A. Määttä[1*]

[1]Institute of Biomedicine, Faculty of Medicine, University of Turku, Kiinamyllynkatu 10, 20520 Turku, Finland

[2]Department of Biomaterials Science and Turku Clinical Biomaterials Centre – TCBC, Institute of Dentistry, University of Turku, Lemminkäisenkatu 2, 20520 Turku, Finland

[3] Wellbeing Services County of Southwest Finland

Corresponding authors:

Karoliina Kajander, E-mail address: kamasuo@utu.fi

Jorma Määttä, E-mail address: jmaatta@utu.fi

Supplementary Figures and Tables

Supplementary Table S1. The total number of cells from the migration experiment end time points in biomaterial and control groups.

Time (h)	Biomaterial					
	Control	Al2O3	BG 45S5	BG S53P4	HAP	CAP
6	249 ± 111	194 ± 32	299 ± 97	300 ± 103	259 ± 83	233 ± 94
12	274 ± 91	392 ± 148	463 ± 182*	499 ± 201*	377 ± 185	206 ± 45
24	457 ± 132	571 ± 175	672 ± 189*	618 ± 272	601 ± 300*	301 ± 87*
48	968 ± 313	1146 ± 528	1202 ± 426*	1210 ± 448	1071 ± 279	632 ± 435

Biological repeats n=3 and technical repeats n=3. Data are presented as mean ±standard deviation. HAP, hydroxyapatite; CAP, carbonated apatite. Statistical significances compared to control within each time point are referred to as *$p \leq 0.05$.

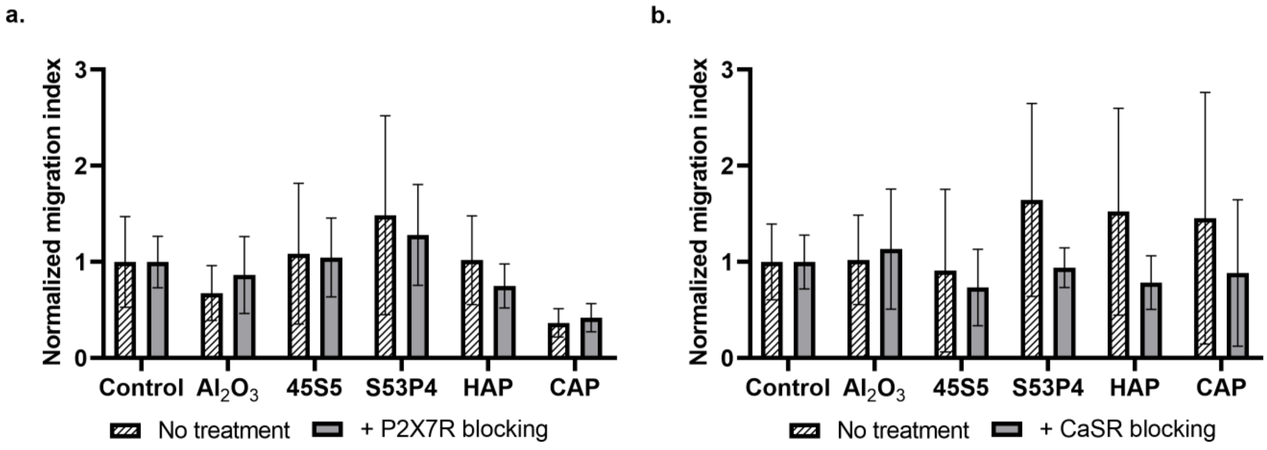

Supplementary Figure S1. The effects of calcium related receptor blocking on cell migration in the Boyden chamber assay after 12 hours. (a) Number of migrated cells after ionotropic P2X7 receptor blocking. (b) Number of migrated cells after calcium sensing receptor (CaSR) blocking. Results are normalized to control group and all data is presented as the mean ±standard deviation from two independent experiments. HAP, hydroxyapatite; CAP, carbonated apatite.

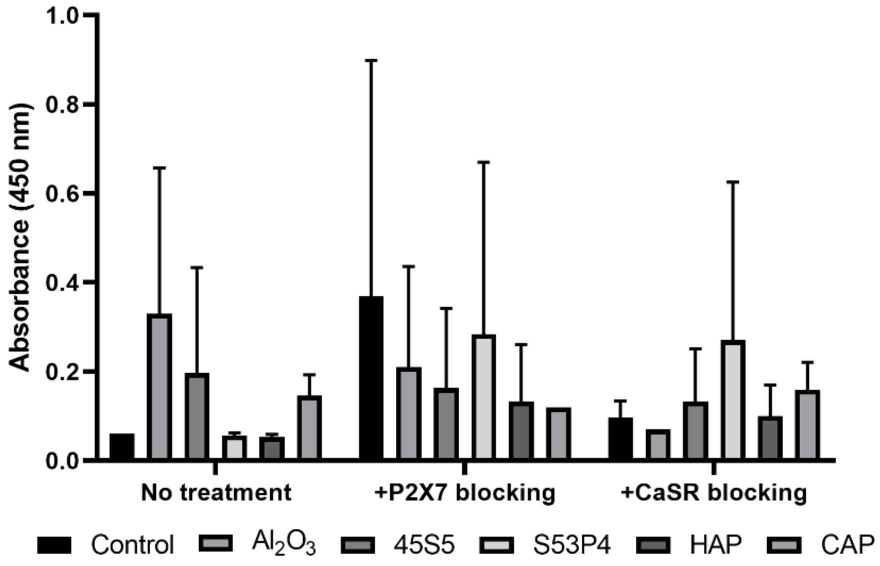

Supplementary Figure S2. The effects of P2X7 or calcium sensing receptor (CaSR) inhibition on osteopontin secretion from MC3T3-E1 cells in response to different biomaterials after 12 hours. Results are presented as the mean ±standard deviation from one experiment. Since the absolute osteopontin concentration in several samples was close to or even below the assay detection limit, the data is shown as absorbance unit at 450 nm. HAP, hydroxyapatite; CAP, carbonated apatite.

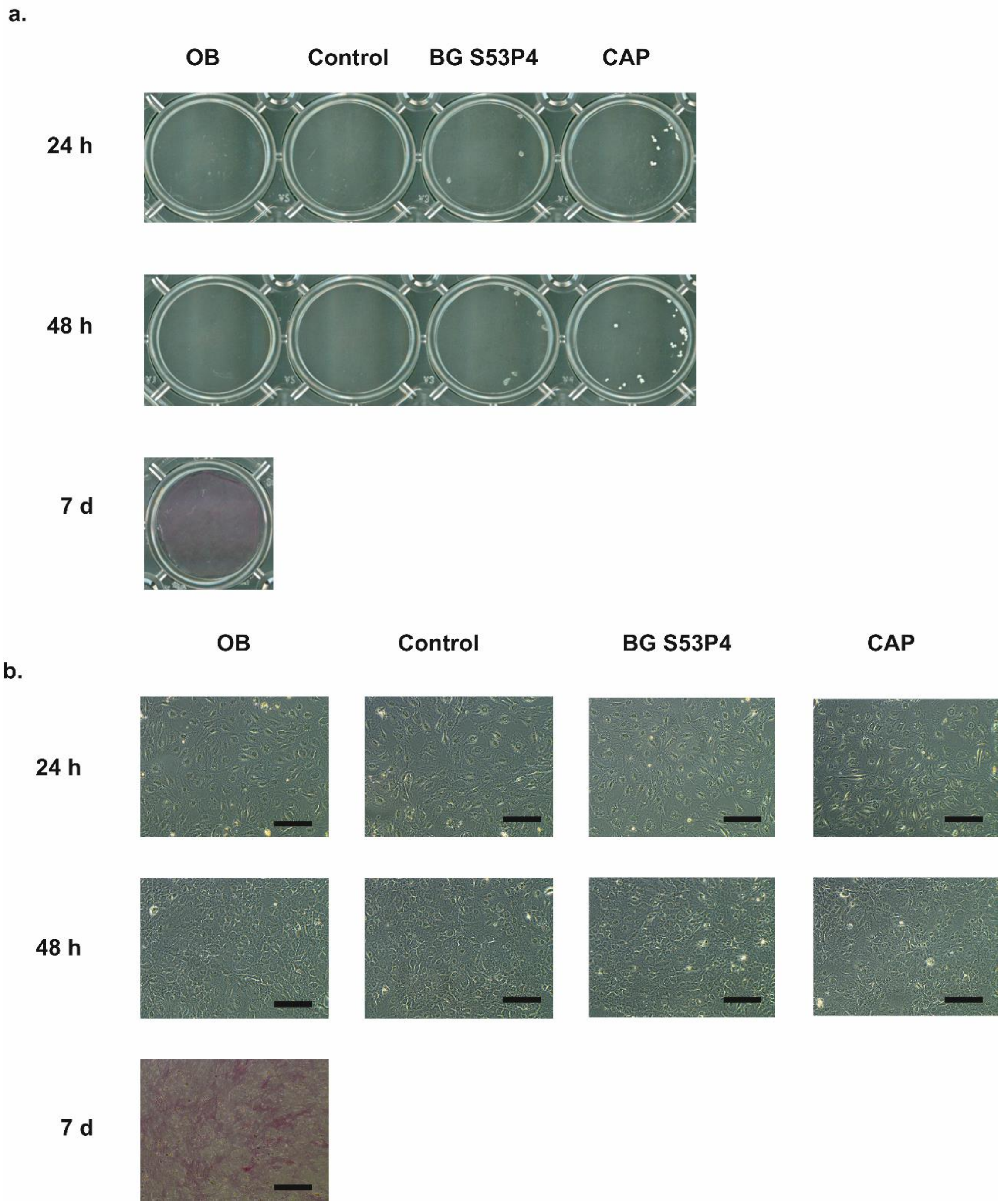

Supplementary Figure S3. Alkaline phosphatase (ALP) staining of MC3T3-E1 cells grown either alone (either in osteoblastic differentiation inducing media, OB, or in normal growth medium, control) or together with 15 mg of bioactive glass (BG) S53P4 or carbonated apatite (CAP). Representatives pictures of stained 24-well culture plates (a) and with 10X magnification (b.). Scale bar is 200 µm.

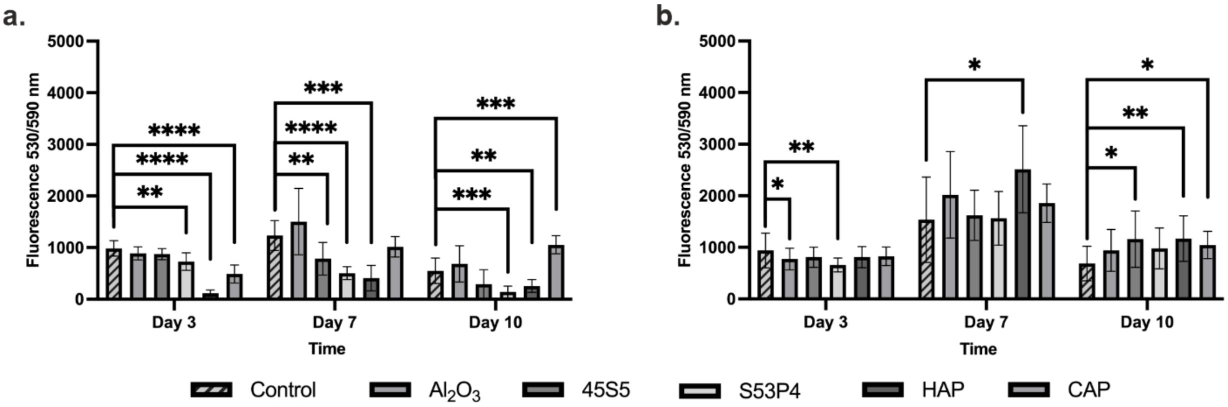

Supplementary Figure S4. The effects of different biomaterials on the viability of pre-osteoblastic MC3T3-E1 cells in media containing 0.5% FBS as measured by Alamar Blue. (a) Cell viability in the presence of biomaterials. (b) Cell viability in the presence of biomaterial-conditioned media. Data is presented as the mean ±standard deviation from three independent experiments. Statistical significances were calculated in GraphPad Prism. Non-parametric data was analyzed using Mann-Whitney test. Statistical significances are referred to as $*p \leq 0.05$, $**p \leq 0.01$, $***p \leq 0.001$ and $****p \leq 0.0001$. HAP, hydroxyapatite; CAP, carbonated apatite.

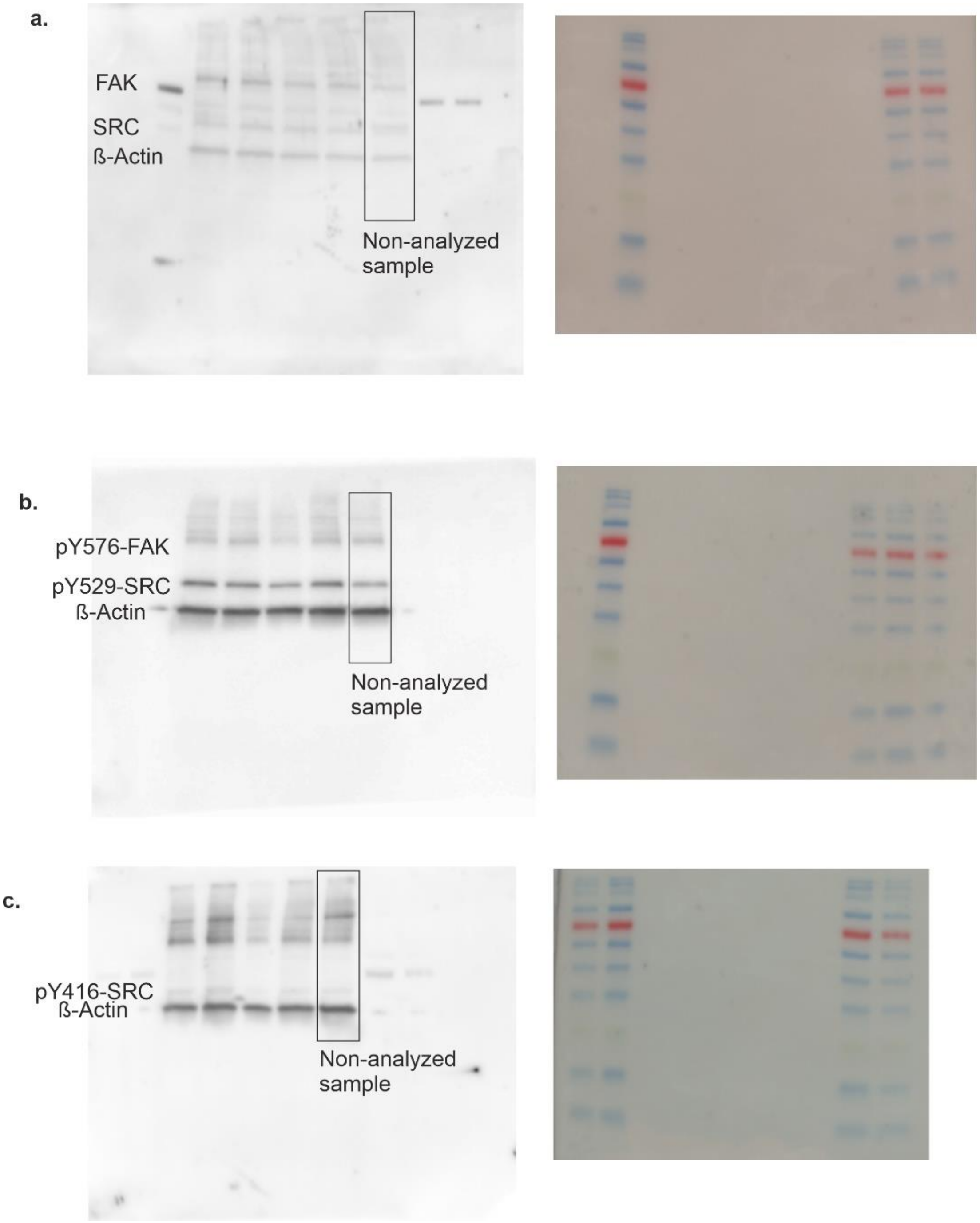

Supplementary Figure S5. Full-length images of Western blot membranes. (a) The blot used for Fig 1b. (b) The blot used for Fig 1c. (c) The blot used for Fig 1d. The protein markers from each membrane are presented on the right side of each blot image.
